# Association of cardiac injury with hypertension in hospitalized patients with COVID-19 in China

**DOI:** 10.1038/s41598-021-01796-0

**Published:** 2021-11-17

**Authors:** Xiaofang Zeng, Anandharajan Rathinasabapathy, Dongliang Liu, Lihuang Zha, Xiangwei Liu, Yiyang Tang, Famei Li, Wenchao Lin, Zaixin Yu, Huiling Chen

**Affiliations:** 1 Department of Cardiology, Xiangya Hospital, Central South University, Changsha, China; 2 National Clinical Research Center for Geriatric Disorders, Xiangya Hospital, Central South University, Changsha, China; 3 Division of Allergy, Pulmonary, and Critical Care Medicine, Vanderbilt University Medical Center, Nashville, TN USA; 4 Department of Spine Surgery, Xiangya Hospital, Central South University, Changsha, China; 5 Department of Geriatric, Xiangya Hospital, Central South University, 87 Xiangya Road, Changsha, 410008 Hunan China

**Keywords:** Hypertension, Prognosis

## Abstract

Outbreak of global pandemic Coronavirus disease 2019 (COVID-19) has so far caused countless morbidity and mortality. However, a detailed report on the impact of COVID-19 on hypertension (HTN) and ensuing cardiac injury is unknown. Herein, we have evaluated the association between HTN and cardiac injury in 388 COVID-19 (47.5 ± 15.2 years) including 75 HTN and 313 normotension. Demographic data, cardiac injury markers, other laboratory findings, and comorbidity details were collected and analyzed. Compared to patients without HTN, hypertensive-COVID-19 patients were older, exhibited higher C-reactive protein (CRP), erythrocyte sedimentation rate, and comorbidities such as diabetes, coronary heart disease, cerebrovascular disease and chronic kidney disease. Further, these hypertensive-COVID-19 patients presented more severe disease with longer hospitalization time, and a concomitant higher rate of bilateral pneumonia, electrolyte disorder, hypoproteinemia and acute respiratory distress syndrome. In addition, cardiac injury markers such as creatine kinase (CK), myoglobin, lactic dehydrogenase (LDH), and N-terminal pro brain natriuretic peptide were significantly increased in these patients. Correlation analysis revealed that systolic blood pressure correlated significantly with the levels of CK, and LDH. Further, HTN was associated with increased LDH and CK-MB in COVID- 19 after adjusting essential variables. We also noticed that patients with elevated either high sensitivity-CRP or CRP demonstrated a significant high level of LDH along with a moderate increase in CK (*p* = 0.07) and CK-MB (*p* = 0.09). Our investigation suggested that hypertensive patients presented higher risk of cardiac injury and severe disease phenotype in COVID-19, effectively control blood pressure in HTN patients might improve the prognosis of COVID-19 patients.

## Introduction

The outbreak of novel coronavirus disease 2019 (COVID-19), set by severe acute respiratory syndrome coronavirus type 2 (SARS-CoV-2) has transformed into a global pandemic since it emerged in Wuhan, China in December 2019^1^. Accruing data from the Center for Systems Science and Engineering at Johns Hopkins University has reported over 200 million confirmed COVID-19 cases and 4 million deaths global wide, till date (https://coronavirus.jhu.edu/map.html). SARS-CoV-2 is much more contagious than SARS-CoV and Middle East Respiratory Syndrome Coronavirus because of its mutation in the receptor binding domain and the subsequent acquisition of furin cleavage site in the S spike protein^2,3^. Although the vaccine is available, the emergence of SARS-CoV-2 variants, such as SARS-CoV-2 spike protein substitution D614G, are still a threat for the global health and economy^4,5^.

Recent investigation demonstrates that cardiovascular comorbidities increase the susceptibility and severity of COVID-19 and also associated with poor disease prognosis^6^. It is well understood that both SARS-CoV and SARS-CoV-2 use angiotensin-converting enzyme 2 (ACE2) as a receptor to gain entry into the host system^7^. Physiologically, ACE2 acts as a potent vasodilator converting Angiotensin II (Ang II) to Angiotensin 1–7 and hence, negatively regulates renin–angiotensin–aldosterone (RAAS) and renin–angiotensin system (RAS) system^8^. Ang II is a potent vasoconstricting octapeptide hormone that plays a pivotal role in the pathogenesis of hypertension (HTN)^9,10^. HTN has been recently identified as the most frequent comorbidity in individuals with COVID-19^11,12^ and it increases the odds ratio for death by 3.05^13^. Further, patients with pre-existing HTN represent a large proportion of patients who incur severe COVID-19 disease symptoms and disproportionately experience worse outcome than one without HTN^13–15^.

Cardiac injury occurs at an early stage of COVID-19, such that rate of incidence noticed during admission at 16.44% rose up to 25.53% with those non-survivors^16^. Similarly, another study with a big cohort of 416 patients reported a 19.7% incidence of cardiac injury in COVID-19 patients. These reports suggest that concurrent myocardial damage could be an independent risk factor in hospital mortality^12^. Although a precise underlying mechanism driving cardiac injury in COVID-19 remains obscure, plausible reasons could be viral infection or a preexisting cardiovascular disease or the combination of both and this is worsened in the patients, who have an exaggerated cytokine storm^4^. However, two recent independent postmortem examinations demonstrated the absence of viral particles in cardiac tissues irrespective of a pronounced local tissue inflammation^17,18^ which suggests that deteriorating pre-existing cardiovascular disease could be the primary cause of cardiac injury in COVID-19 patients. Since ACE2 is extensively expressed in the heart tissue and plays a role in cardiovascular homeostasis, we decided to investigate the role of HTN in SARS-CoV-2 mediated myocardial damage in COVID-19 patients. As determined, this study examined in depth on the potential association between HTN and cardiac injury in patients with COVID-19.

## Methods

### Study design and participants

In this multicenter retrospective study, 388 laboratory-confirmed COVID-19 patients admitted in the designated hospitals between January 16^th^ and March 14^th^, 2020 were randomly enrolled. Patients, who were 18 years or older were included in this investigation. The diagnosis for COVID-19 was strictly followed in accordance with World Health Organization interim guidance^19^ and the principles outlined in the Declaration of Helsinki. This study protocol was approved by the Ethics Committee of Xiangya Hospital, Central South University, Hunan and the informed written consent was obtained from all study participants.

### Data collection

The medical records of 388 COVID-19 patients were retrospectively reviewed and analysed. Epidemiological (age and sex) as well as clinical information (symptoms, chronic medical histories, laboratory findings and other complications) along with the radiological results, documented during hospitalization were collected and verified independently by two physicians. Based on the seventh edition of the Chinese National Health Commission (https://www.scirp.org/reference/referencespapers.aspx?referenceid=2851516), patients were categorized into severe disease status, if they meet any of the following criteria: 1) shortness of breath, respiratory rate ≥ 30 beats per min; 2) oxygen saturation ≤ 93% at rest; 3) arterial oxygen partial pressure/oxygen concentration ≤ 300 mmHg (1 mmHg = 0.133 kPa); 4) lung images showing obvious progress of lesion size > 50% within 24 − 48 h. and patients were classified into mild disease status, if they presented mild clinical symptoms or mild/no lesions on imaging findings.

Cardiac injury biomarker data such as creatine kinase (CK), creatine kinase-MB (CK-MB), myoglobin, lactate dehydrogenase (LDH), and N-terminal pro brain natriuretic peptide (NT-proBNP) were assessed immediately after hospitalization. Cardiac troponin I (cTnI) was not accounted in this study, since most of the patients presented within the normal range of cTnI and that was indicated as < 0.01 ng/mL. Criteria of acute respiratory distress syndrome (ARDS) was followed as mentioned in the Berlin definition^18^. All patients were carefully reviewed for their chronic medical history (coronary heart disease, arrhythmia, hyperlipidaemia, HTN diabetes, chronic obstructive pulmonary disease, chronic kidney disease, chronic liver disease, and cerebrovascular disease) and divided into two groups based on their pre-existing HTN. All clinical features and study outcomes were compared between hypertensive- and normotensive-COVID-19 patients.

Subgroup analysis on the correlation between inflammation and cardiac injury was conducted in COVID-19 patients concurrent with HTN. Specifically, hypertensive COVID-19 patients were divided into two groups based on their elevated level of either C-reactive protein (CRP) or high sensitivity-CRP (hs-CRP), and cardiac injury markers were compared.

### Statistical analysis

Statistical analyses were performed using SPSS version 20.0 statistical software (SPSS Inc., Chicago, IL, USA). Descriptive statistics was obtained for all study variables. Continuous variables were presented as mean ± standard deviation (SD), if they were normally distributed; otherwise, they were presented as median ± range interquartile (IQR), and categorical variables as frequencies. All categorical variables between the patients with and without HTN were analyzed using Fisher exact test or χ2 test and continuous variables were compared using t-test or Mann–Whitney U test, as appropriate. The Spearman correlation coefficient was calculated to disclose relationship of inflammatory or cardiac injury markers with blood pressure. Further, binary logistic regression model was performed to determine the association between blood pressure and cardiac injury in these patients after adjusting essential variables. Statistical charts were generated using Prism 7 (GraphPad Software Inc., San Diego, CA, USA). A two-sided *p* < 0.05 was considered statistically significant for the entire data set.

## Results

### Patients characteristics

Our investigation presented a total of 388 hospitalized COVID-19 patients (197 male and 191 female) with an average age, 47.5 ± 15.2 years. Fever and cough were the most common symptoms observed in 227 (58.4%) and 217 (55.8%) patients, followed by fatigue, sputum production and shortness of breath 67 (17.2%), 56 (14.4%) and 36 (9.3%), respectively. Symptoms such as muscle ache, diarrhea, chest tightness, sore throat, rhinorrhea and headache were rare and noted in 16 (4.1%), 10 (2.6%), 7 (1.8%), 15 (3.9%), 10 (2.6%) and 16 (4.1%) patients, respectively. We also noticed several other comorbidities such as diabetes, chronic liver disease, coronary heart disease, chronic obstructive pulmonary disease, hyperlipidemia, cerebrovascular disease, chronic kidney disease and arrhythmia in at least 50 (12.9%), 27 (6.9%), 22 (5.7%), 22 (5.7%), 13 (3.3%), 13 (3.3%), 10 (2.6%) and 3 (0.8%) patients, respectively. Rest of the baseline characteristics are outlined in the Table 1.

**Table 1:** Baseline characteristics of patients infected with COVID-19.

Variables	Total (n = 388)	Normotension (n = 313)	Hypertension (n = 75)	*p* value
Age (mean, year)	47.5 ± 15.2	44.5 ± 11.9	60.2 ± 14.2	< 0.001
Gender (male), n (%)	197 (50.8%)	159 (50.8)	38 (50.7)	0.984
SBP (mean, mmHg)	126.6 ± 14.8	123.8 ± 13.2	138.5 ± 15.2	< 0.001
DBP ( mean, mmHg)	79.7 ± 10.9	78.2 ± 10.2	86.2 ± 11.4	< 0.001
Temperature (mean, ℃)	37.0 ± 0.69	37.0 ± 0.7	36.9 ± 0.8	0.498
Heart rate (mean, bpm)	86.7 ± 12.7	86.0 ± 12.1	89.8 ± 14.9	≤ 0.05
**Symptoms (n, %)**				
Fever	227 (58.4)	126 (40.3)	40 (53.3)	≤ 0.05
Cough	217 (55.8)	173 (55.3)	44 (58.7)	0.361
Fatigue	67 (17.2)	256 (81.8)	10 (13.3)	< 0.001
Sputum production	56 (14.4)	7 (2.2)	9 (12.0)	< 0.001
Shortness of breath	36 (9.3)	4 (1.3)	13 (17.3)	< 0.001
Muscle ache	16 (4.1)	13 (4.2)	3 (4.0)	0.952
Diarrhea	10 (2.6)	8 (2.6)	2 (2.7)	0.957
Chest tightness	7 (1.8)	4 (1.3)	3 (4.0)	0.135
Sore throat	15 (3.9)	14 (4.5)	1 (1.3)	0.321
Rhinorrhea	10 (2.6)	7 (2.2)	1 (1.3)	0.621
Headache	16 (4.1)	14 (4.5)	2 (2.7)	0.747
**Any comorbidity (n, %)**				
Diabetes	50 (12.9)	27 (8.6)	23 (30.7)	< 0.001
Chronic liver disease	27 (6.9)	23 (7.3)	4 (5.3)	0.800
Coronary heart disease	22 (5.7)	6 (1.9)	16 (21.3)	< 0.001
Chronic obstructive pulmonary disease	22 (5.7)	15 (4.8)	7 (9.3)	0.160
Hyperlipidaemia	13 (3.3)	11 (3.5)	2 (2.7)	0.957
Cerebrovascular disease	13 (3.3)	6 (1.9)	7 (9.3)	< 0.005
Chronic kidney disease	10 (2.6)	5 (1.6)	5 (6.7)	≤ 0.05
Arrhythmia	3 (0.8)	2 (0.6)	1 (1.3)	0.476

Although the mean systolic and diastolic blood pressure and heart rate of entire study subjects were in the normal range (systolic blood pressure, 126.6 ± 14.8 mmHg, diastolic blood pressure, 79.7 ± 10.9 mmHg and heart rate, 86.7 ± 12.7 beats per minute), a detailed analysis revealed that 75 (19.3%) patients were hypertensives (Table 1). A systemic comparison of normotensive COVID-19 patients with the hypertensives demonstrated that later were older (44.5 ± 11.9 *vs.* 60.2 ± 14.2, *p* < 0.001) and presented higher heart rate (86.0 ± 12.1 *vs.* 89.8 ± 14.9, *p* < 0.05), incidence of fever (40.3% *vs.* 53.3%, *p* < 0.05), sputum production (2.2% *vs.* 12.0%, *p* < 0.001) and shortness of breath (1.3% *vs.* 17.3%, *p* < 0.001). In addition, increased frequency was noticed for comorbidity such as diabetes (8.6 vs. 30.7%, *p* < 0.001), coronary heart disease (1.9 vs. 21.3%, *p* < 0.001), cerebrovascular disease (1.9 vs. 9.3%, *p* < 0.001) and chronic kidney disease (1.6 vs. 6.7%, *p* < 0.05) in hypertensive-COVID-19 patients. Surprisingly, neither the mean temperature of whole study subjects nor the normotensive *vs*. hypertensive groups were different from one other (Table 1).

### Laboratory and radiographic findings

We next sought to investigate the metrics of laboratory and radiography assessments. As expected, hypertensive-COVID-19 patients demonstrated a significant higher level of CRP ( 12.45[5.00–38.60] *vs.* 9.30[2.31–23.68], *p* < 0.05), hs-CRP (12.34[4.20–37.13] *vs.* 5.38[0.50–22.48], *p* < 0.05), erythrocyte sedimentation rate (46.00[25.25, 70.00] *vs.* 38.00[18.00–60.00], *p* < 0.05) and alanine aminotransferase (26.65[16.00–42.25] *vs.* 24.00[16.00–34.00], *p* < 0.05) (Table 2). Other biochemical and blood factors such as creatinine, uric acid, urea nitrogen, white blood cells and neutrophils demonstrated an increasing trend in the hypertensives, although those were statistically insignificant. Rest of the laboratory metrics such as lymphocytes, platelets, erythrocytes, hemoglobin, electrolyte ions, lipid molecules and etc., demonstrated a subtle or no change (Table 2). Further, radiography assessments demonstrated an increased prevalence of bilateral pneumonia (85.6 *vs*. 97.3%, *p* < 0.001) in patients with HTN.

**Table 2:** Laboratory findings of patients infected with COVID-19 on admission.

Variables, median (IQR)	Total (n = 388)	Normotension (n = 313)	Hypertension (n = 75)	*p* value
White blood cells	4.77 (3.70–6.26)	4.65 (3.65–6.26)	5.05 (3.89–6.35)	0.443
Neutrophil	3.14 (2.32–4.47)	3.10 (2.26–4.41)	3.44 (2.40–4.59)	0.624
Lymphocyte	1.07 (0.77–1.46)	1.06 (0.78–1.48)	1.10 (0.74–1.34)	0.446
Platelets	178.00 (142.00–225.00)	178.00 (142.99–225.50)	178.00 (135.00–223.00)	0.509
Haemoglobin	132.50 (121.00–143.00)	133.00 (122.00–144.00)	129.00 (121.00–139.00)	0.708
CRP	9.65 (2.98–26.70)	9.30 (2.31–23.68)	12.45 (5.00–38.60)	≤ 0.05
Hs-CRP	6.00 (0.61–23.75)	5.38 (0.50–22.48)	12.34 (4.20–37.13)	0.03
K^+^	3.96 (3.62–4.25)	3.95 (3.63–4.25)	3.98 (3.59–4.28)	0.762
Na^+^	138.80 (136.50–140.60)	138.70 (136.50–140.40)	138.85 (136.30–140.85)	0.554
Cl^−^	103.15 (100.00–105.30)	103.40 (100.23–105.40)	101.85 (99.10–104.65)	0.195
Ca^+^	2.22 (2.11–2.36)	2.23 (2.11–2.34)	2.22 (2.10–2.42)	0.178
ESR (mm/h)	40.00 (20.00–64.00)	38.00 (18.00–60.00)	46.00 (25.25,70.00)	≤ 0.05
Total protein, (g/L)	69.50 (64.80–74.10)	74.40 (65.10–74.65)	66.25 (63.68–71.70)	0.381
Albumin, g/L	40.50 (37.12–44.40)	40.80 (37.90–44.63)	38.95 (34.45–42.05)	0.382
Activated partial thromboplastin time, s	31.80 (27.80–36.43)	32.05 (28.03–36.85)	30.30 (26.80–35.35)	0.325
D-dimer, mg/L	0.35 (0.20–0.59)	0.33 (0.18–0.59)	0.42 (0.27–0.67)	0.381
Fibrinogen (g/L)	3.69 (2.86–4.71)	3.67 (2.75–4.72)	4.00 (3.10–4.66)	0.461
Alanine aminotransferase, U/L	24.00 (19.00–34.00)	24.00 (19.00–32.00)	27.50 (20.8–43.50)	≤ 0.05
Aspartate aminotransferase, U/L	24.00 (16.00–35.00)	24.00 (16.00–34.00)	26.65 (16.00–42.25)	0.184
Total bilirubin, mmol/L	10.81 (7.90–17.60)	11.15 (7.75–17.30)	10.60 (8.24–18.46)	0.747
Creatinine, μmol/L	64.44 (54.5–77.90)	63.00 (53.78–75.20)	72.00 (57.25–86.45)	0.061
Uric acid (umol/L)	266.62 (206.55–323.25)	258.93 (204.40–318.27)	296.00 (232.00–349.35)	0.114
Urea nitrogen (mmol/L)	3.74 (3.00–4.80)	3.60 (2.90–4.42)	4.43 (3.42–5.81)	0.258
Glucose (mmol/L)	6.30 (5.44–7.81)	6.24 (5.41–7.69)	6.51 (5.57–8.95)	0.815
Cholesterol	3.90 (3.41–4.59)	3.90 (3.33–4.51)	4.02 (3.55–4.76)	0.133
Triglyceride	1.23 (0.92–1.96)	1.22 (0.88,1.89)	1.32 (0.96–2.38)	0.209
HDL	1.14 (0.95–1.33)	1.13 (0.94–1.33)	1.15 (1.01–1.34)	0.724
LDL	2.15 (1.77–2.68)	2.17 (1.74–2.67)	2.12 (1.82–2.78)	0.267
**Chest radiographs and CT finding**				
Unilateral pneumonia	10 (2.6)	10 (3.2)	0 (0)	0.220
Bilateral pneumonia	341 (87.7)	268 (85.6)	73 (97.3)	< 0.001
Normal	16 (4.1)		2 (2.7)	0.747

*CRP* C-reactive protein; *ESR* erythrocyte sedimentation rate; *HDL* high-density lipoprotein; *hs-CRP* high sensitivity C-reactive protein; *IQR* interquartile range; *LDL* low density lipoprotein.

### Complications and clinical outcome

Although the presence of HTN subtly altered the meantime from onset of symptom to hospitalization (5.3 ± 3.6 *vs*. 5.8 ± 3.8 days, *p* = 0.399), it significantly increased mean hospitalization time (15.9 ± 7.1 *vs*. 18.7 ± 8.0, days, *p* < 0.001). Further, hypertensive-COVID-19 patients displayed a severe disease status (12.1 *vs*. 76.0%, *p* < 0.001) and presented other complications such as electrolyte disorder (11.2 *vs*. 17.3%, *p* < 0.001), hypoproteinemia (4.8 *vs*. 17.3%, *p* < 0.001) and ARDS (2.6 *vs*. 8.0%, *p* < 0.001), while complications such as anemia, pneumonia, arrhythmia and leukopenia were insignificantly altered (Table 3).

**Table 3:** Complications of patients infected with COVID-19.

Variables	Total (n = 388)	Normotension (n = 313)	Hypertension (n = 75)	*p* value
Time of hospitalization, mean (SD), d	16.4 ± 7.3	15.9 ± 7.1	18.7 ± 8.0	< 0.001
Time from symptom onset to hospital admission, mean (SD)	5.4 ± 3.6	5.3 ± 3.6	5.8 ± 3.8	0.399
**Severity of illness (n, %)**				
Severe	56 (11.4)	38 (12.1)	57 (76)	< 0.001
Mild	332 (85.3)	275 (87.9)	18 (24)	
**Complications (n, %)**				
Drug-induced hepatitis	32 (8.2)	26 (8.3)	6 (8.0)	0.931
Electrolyte disorder	48 (12.3)	35 (11.2)	13 (17.3)	≤ 0.05
Hypoproteinemia	28 (7.2)	15 (4.8)	13 (17.3)	< 0.001
Anemia	20 (5.1)	15 (4.8)	5 (6.7)	0.560
Pneumonia	22 (5.7)	16 (5.1)	6 (8.0)	0.401
Arrhythmia	7 (1.8)	6 (1.9)	1 (1.3)	0.998
Leukopenia	8 (2.1)	8 (2.6)	0 (0)	0.363
Acute respiratory distress syndrome	14 (3.6)	8 (2.6)	6 (8.0)	≤ 0.05

### HTN and cardiac injury

**Figure 1:**
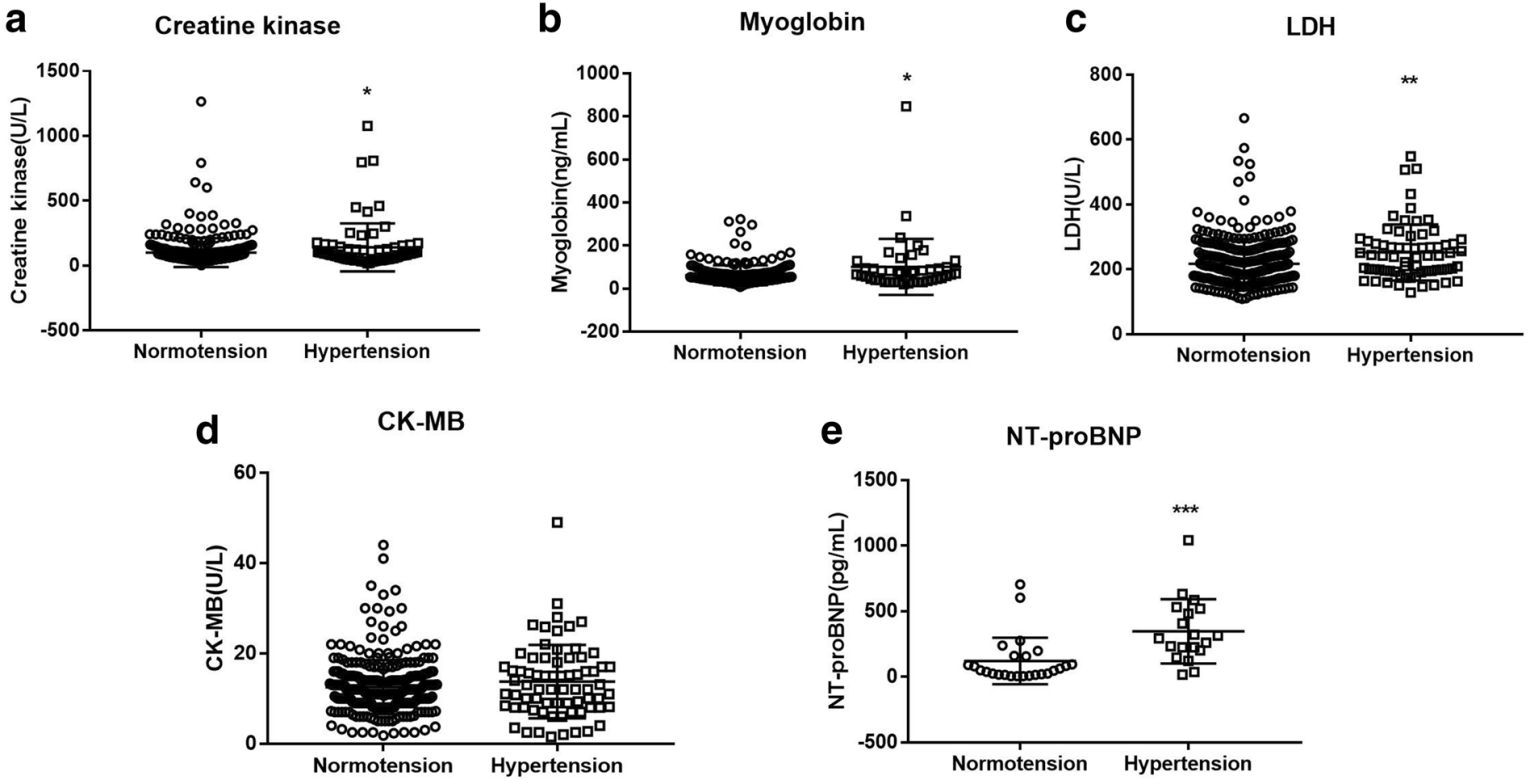
Cardiac injury markers in COVID-19 patients with and without hypertension. Caption: *CK* creatine kinase; *CK-MB* creatine kinase isoenzymes; *LDH* lactate dehydrogenase; *NT-proBNP* N-terminal pronatriuretic peptide. Data was presented as mean ± SD *, *p* < 0.05, **, *p* < 0.01, ***, *p* < 0.001: COVID-19 patients with *vs.* without hypertension.

**Figure 2:**
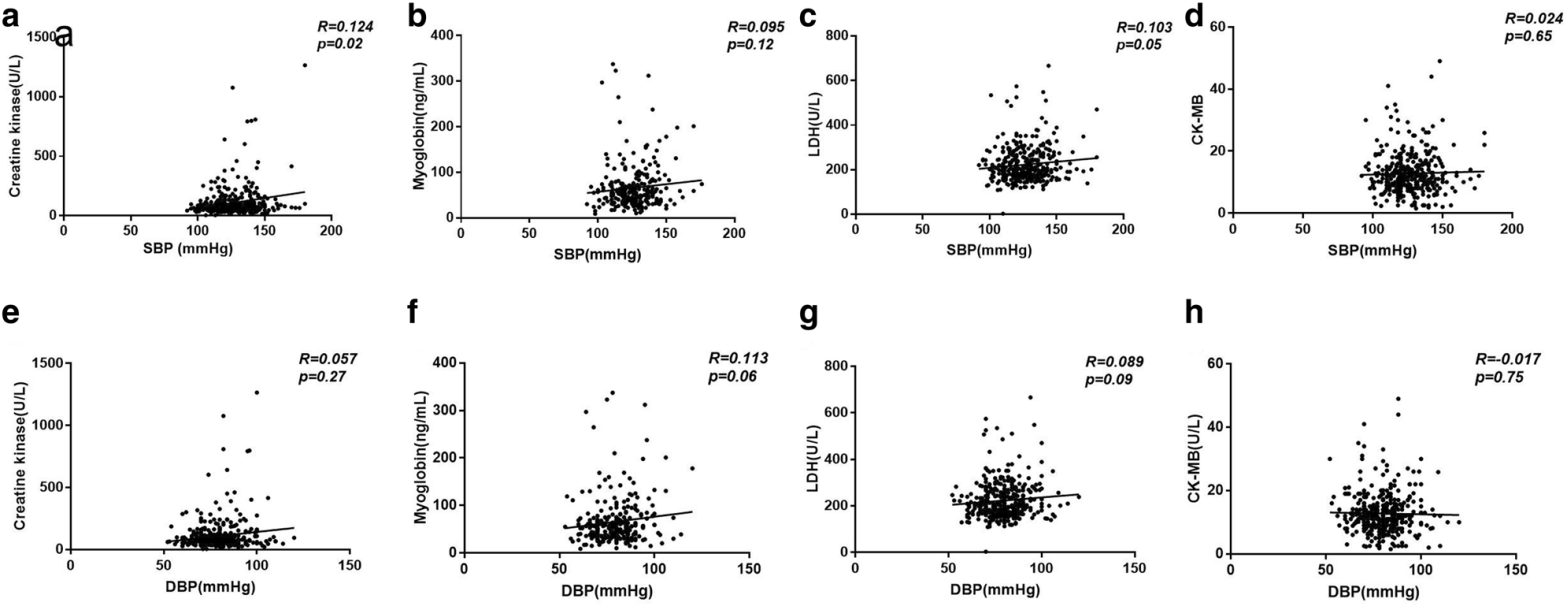
Correlation between blood pressure and cardiac injury markers in COVID-19. Caption: *CK* creatine kinase; *CK-MB* creatine kinase isoenzymes; *DBP* diastolic blood pressure; *LDH* lactate dehydrogenase; *SBP* systolic blood pressure; *NT-proBNP* N-terminal pronatriuretic peptide.

Next, we sought to investigate the association between blood pressure and cardiac injury. As seen in the Fig. 1, patients with HTN demonstrated a significant increase in the level of cardiac injury markers – CK, myoglobin, LDH, and NT-pro BNP, while change in CK-MB was subtle. Correlation analysis revealed that systolic blood pressure in patients with COVID-19 correlated significantly with the levels of CK (R = 0.124, *p* = 0.01), and LDH (R = 0.103, *p* = 0.05); while diastolic blood pressure showed a trend to correlate with the level of myoglobin (R = 0.113, *p* = 0.06) and LDH (R = 0.089, *p* = 0.09) (Fig. 2). Further logistic regression analysis showed that HTN was associated with increased LDH (*p* = 0.001) and CK-MB (*p* = 0.05) in COVID- 19 patients after adjusting age, gender, and comorbidity of diabetes, coronary heart disease, chronic obstructive pulmonary disease, cerebrovascular disease, and chronic kidney disease (Table 4).

**Table 4:** Relationship between hypertension against markers of cardiac injury.

Variable			Crude			Model I	
			OR (95%CIs)		*p* value	OR (95%CIs)	*p* value
**Increased CK (NR:25–200U/L)**							
**Blood pressure**							
Normotension			1 (ref)			1 (ref)	
Hypertension			1.70 (0.80, 3.55)		0.17	1.39 (0.60, 3.25)	0.59
**Increased Myoglobin (NR:0–85 ng/mL)**							
**Blood pressure**							
Normotension			1 (ref)			1 (ref)	
Hypertension			2.75 (1.34, 5.65)		0.006	1.70 (0.76, 3.82)	0.14
**Increased CK-MB (NR:0–24U/L)**							
**Blood pressure**							
Normotension			1 (ref)			1 (ref)	
Hypertension			2.74 (1.09, 6.88)		0.03	2.50 (0.98, 6.39)	0.05
**Increased LDH (NR:95–250U/L)**							
**Blood pressure**							
Normotension			1 (ref)			1 (ref)	
Hypertension			2.64 (1.53, 4.57)		0.001	2.64 (1.53, 4.57)	0.001

Caption: *CK* creatine kinase; *CK-MB* creatine kinase isoenzymes; *LDH* lactate dehydrogenase; Crude model adjusted for none. Model I adjusted for age, gender, and comorbidity of Diabetes, Coronary heart disease, Chronic obstructive pulmonary disease, Cerebrovascular disease, and Chronic kidney disease.

**Figure 3:**
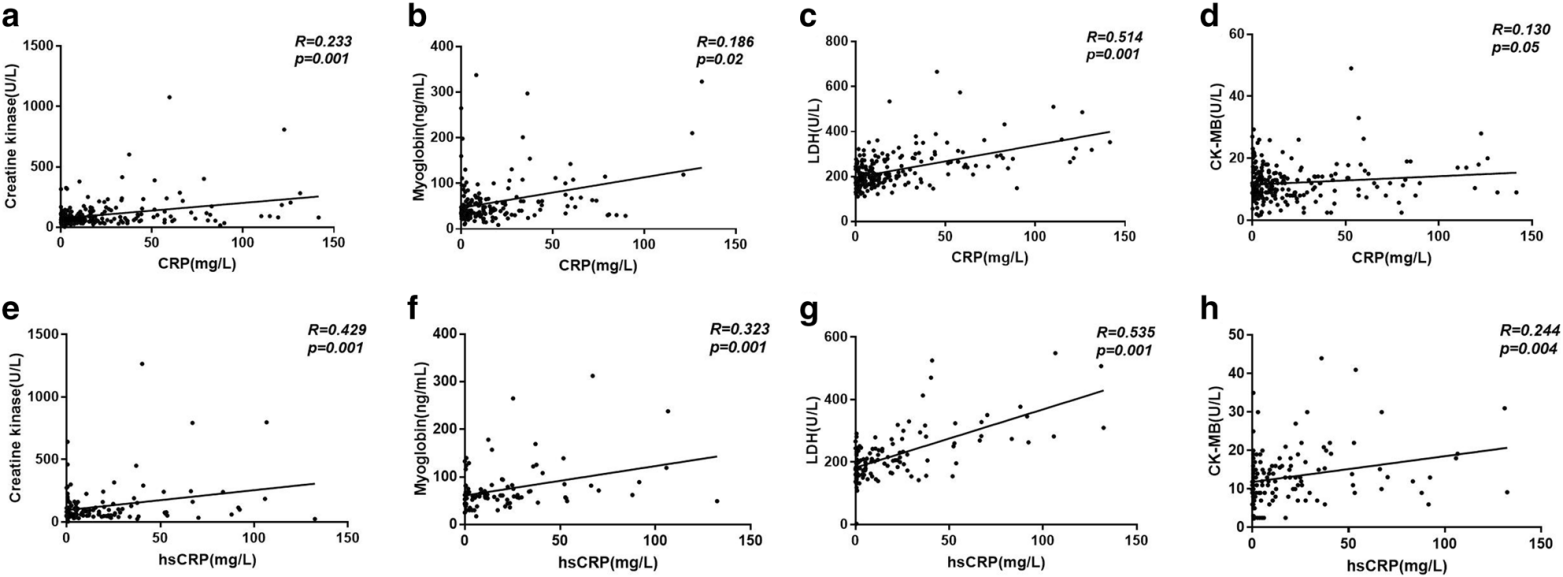
Correlation between CRP/hsCRP and cardiac injury markers in COVID-19. Caption: *CK* creatine kinase; *CK-MB* creatine kinase isoenzymes; *CRP* C-reactive protein; *hsCRP* high sensitive C-reactive protein; *LDH* lactate dehydrogenase; *NT-proBNP* N-terminal pronatriuretic peptide.

**Figure 4:**
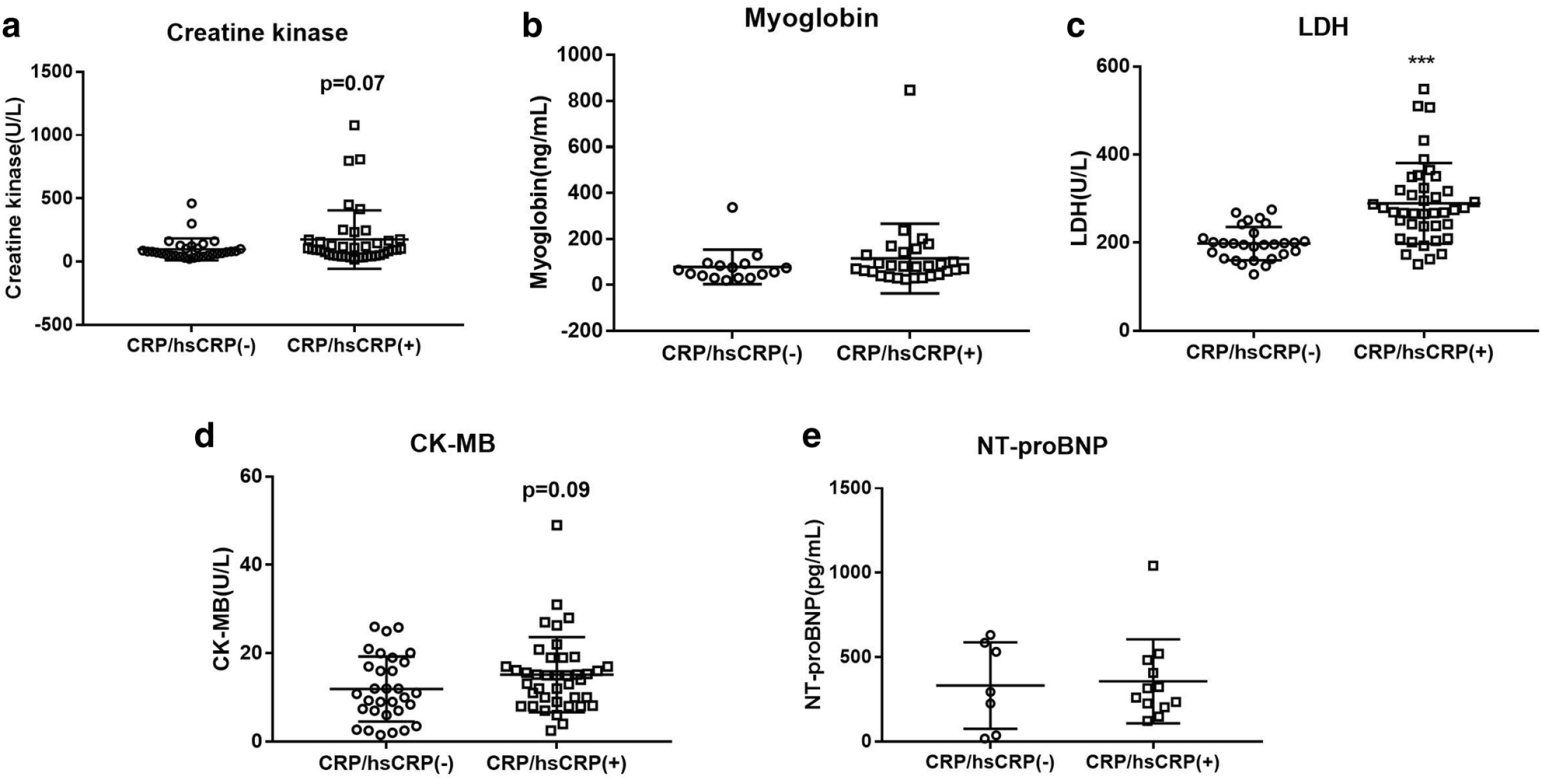
Cardiac injury markers in hypertensive COVID-19 patients with and without increased CRP/hsCRP. Caption: *CK-MB* creatine kinase isoenzymes; *CRP* C-reactive protein; *hsCRP* high sensitive C-reactive protein; *LDH* lactate dehydrogenase; *NT-proBNP* N-terminal pronatriuretic peptide. Data was presented as mean ± SD. ***, *p* < 0.001: COVID-19 patients with *vs.* without hypertension.

**Figure 5:**
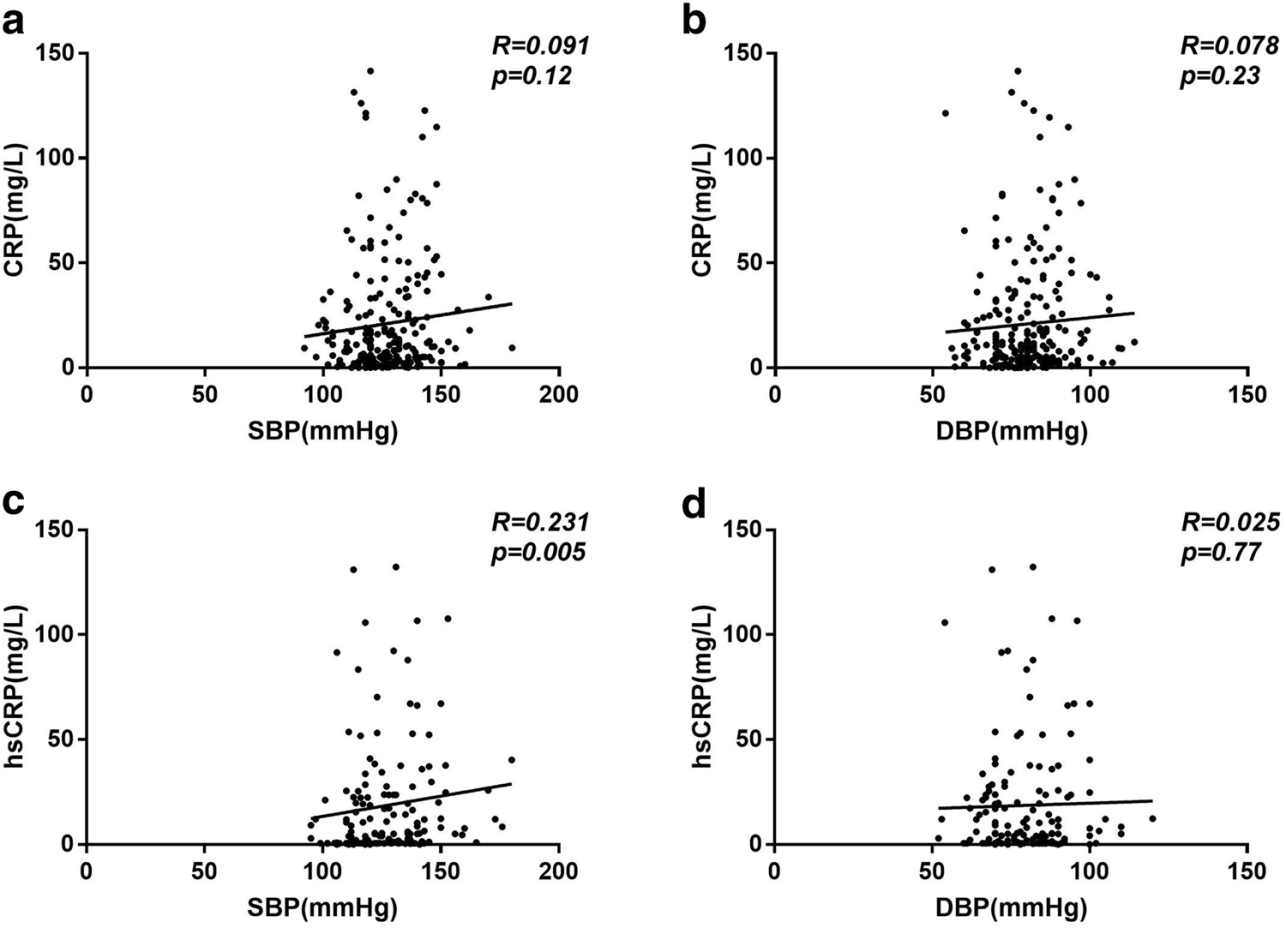
Correlation between blood pressure and CRP/hsCRP in COVID-19. Caption: *CRP* C-reactive protein; *hsCRP* high sensitive C-reactive protein; *DBP* diastolic blood pressure; *SBP* systolic blood pressure.

Previous studies have demonstrated that inflammatory cells such as macrophage and T cells in HTN infiltrate into the heart and results in cardiac and other end-organ damage^20^. A significant correlation between cardiac injury (levels of CK, myoglobin, LDH, and CK-MB) and inflammation (CRP, hs-CRP) has been observed in the present study (Fig. 3). We next compared cardiac injury markers in hypertensive-COVID-19 patients with and without CRP/hsCRP incensement and found that patients who presented an elevated inflammatory status also demonstrated a significant increase in LDH and a moderate increase in CK (*p* = 0.07) and CK-MB (*p* = 0.09) (Fig. 4). Further correlation analysis suggested a significant correlation between hs-CRP and systolic blood pressure in patients with COVID-19 (Fig. 5). Those results suggested that HTN was associated with increased cardiac injury, and inflammation might participate in hypertensive mediated cardiac injury.

## Discussion

To the best of our knowledge, this is the first report providing comprehensive information on epidemiological, demographic, clinical, laboratory and radiological characteristics of hospitalized COVID-19 patients in the context of HTN and ensuing myocardial damage. Our results specifically highlighted a significant association between HTN and cardiac injury in COVID-19 patients. In addition, our subgroup analysis demonstrated that inflammation could be a potential factor driving HTN induced cardiac injury in COVID-19.

The case-fatality rate for overall COVID-19 ranges from 0.0 to 28.9% across different geographical locations, while it rises up to 40% in patients admitted to intensive care unit^21^. Most importantly, cardiac injury has been reported as an independent risk factor of mortality in patients with COVID-19^12,22^. A recent meta-analysis showed that 24.4% of the hospitalized COVID-19 patients developed cardiac injury and 72.6% of them did not survive^23^. Studies also showed that hospitalized, COVID-19 patients with preexisting cardiovascular disease, such as HTN have been more susceptible to COVID-19 related cardiac injury^23,24^. Consistently, our study also demonstrated that HTN was the most common comorbidity (almost 19.3%) observed in hospitalized COVID-19 patients and also, these patients displayed an increase of cardiac injury markers, such as LDH, CK, and myoglobin.

As one of the most common cardiovascular comorbidities, HTN is a risk factor for increased intensive care unit admission^14^, utilization of mechanical ventilation^25^, and mortality^26^. A blood pressure control with a target of < 130/80 mmHg during hospitalization is associated with fewer adverse clinical events in patients with COVID-19^27^. High blood pressure is one of the most critical risk factors for developing cardiovascular complications^28,29^. In this study, we have demonstrated that systolic blood pressure in patients with COVID-19 correlated significantly with the levels of CK, LDH; and hypertension was associated with increased LDH and CK-MB in COVID- 19 patients after adjusting age, gender, and comorbidity of diabetes, coronary heart disease, chronic obstructive pulmonary disease, cerebrovascular disease, and chronic kidney disease. Thus, our study provided direct evidence in establishing a strong association between HTN and cardiac injury in COVID-19.

COVID-19 infection activates an excess production of inflammatory cytokines (interleukin-6 and tumour necrosis factor-α)—a cytokine storm which results in systemic inflammation, multi organ dysfunction and acutely, affecting the cardiovascular system^30,31^. An uncontrolled and dysfunctional immune response characterized by continuous activation and proliferation of lymphocytes and macrophages are the hallmark of this cytokine storm syndrome^32^. In line with this, several autopsy reports have demonstrated an exaggerated infiltration of mononuclear inflammatory cells into the myocardia of COVID-19 patients, who presented higher viral load^18,33,34^.

In this study, we found inflammatory markers such as CRP, hs-CRP were significantly increased in hypertensive-COVID-19. Further, in the hypertensive group, COVID-19 patients with increased CRP or hs-CRP presented an increased level of cardia injury markers, LDH (*p* < 0.001), CK (*p* = 0.07) and CK-MB (*p* = 0.09). Incidentally, chronic inflammation associated with HTN synergizes cytokine storm and worsens host immune surveillance in SARS-CoV-2 infection^35,36^. It has been established that macrophages and T cells infiltrate into the heart in response to HTN, which ultimately results in end-organ damage^20^. Further, the deficiency of CD8 + cytotoxic T cells effectively protect against hypertension-induced cardiac damage in COVID-19^37,38^. A significant linear correlation between hs-CRP and systolic blood pressure has been observed in the present study, suggesting HTN associated pro- inflammatory state might drives cardiac injury that worsens the prognosis of patients with COVID-19.

We acknowledge that number of confirmed COVID-19 patients enrolled in this study is a limitation and further validation of our findings on a larger, multiple centered cohorts is warranted. Moreover, constrains such as space, time and the hospitals designated warded us off from utilizing conventional techniques such as echocardiography and electrocardiography.

## Conclusion

Our investigation suggested that HTN is associated with a higher risk of cardiac injury and severe disease phenotype in COVID-19, effectively control blood pressure in HTN patients might improve the prognosis of COVID-19 patients. This study also suggested HTN associated pro- inflammatory state might drive cardiac injury that worsens the prognosis of patients with COVID-19, thus provided a cue in understanding the mechanism of cardiac injury in COVID-19.

## Data Availability

The datasets generated during and/or analyzed during the current study are available from the corresponding author on reasonable request.
